# Neuritogenic and Neuroprotective Effects of Polar Steroids from the Far East Starfishes *Patiria pectinifera* and *Distolasterias nipon*

**DOI:** 10.3390/md11051440

**Published:** 2013-05-03

**Authors:** Natalia V. Palyanova, Tatyana M. Pankova, Marina V. Starostina, Alla A. Kicha, Natalia V. Ivanchina, Valentin A. Stonik

**Affiliations:** 1 Institute of Molecular Biology and Biophysics, SB RAMS, Novosibirsk 630117, Russia; E-Mails: natasha@soramn.ru (N.V.P.); pan@soramn.ru (T.M.P.); marina@soramn.ru (M.V.S.); 2 G.B. Elyakov Pacific Institute of Bioorganic Chemistry, FEB RAS, Vladivostok 690022, Russia; E-Mails: ivanchina@piboc.dvo.ru (N.V.I.); stonik@piboc.dvo.ru (V.A.S.)

**Keywords:** steroid, starfish, neuroblastoma, organotypic hippocampal slice culture, neuritogenesis, oxygen-glucose deprivation

## Abstract

The neuritogenic and neuroprotective activities of six starfish polar steroids, asterosaponin Р_1_, (25*S*)-5α-cholestane-3β,4β,6α,7α,8,15α,16β,26-octaol, and (25*S*)-5α-cholestane-3β,6α,7α,8,15α,16β,26-heptaol (**1**–**3**) from the starfish *Patiria pectinifera* and distolasterosides D_1_–D_3_ (**4**–**6**) from the starfish *Distolasterias nipon* were analyzed using the mouse neuroblastoma (NB) C-1300 cell line and an organotypic rat hippocampal slice culture (OHSC). All of these compounds enhanced neurite outgrowth in NB cells. Dose-dependent responses to compounds **1**–**3** were observed within the concentration range of 10–100 nM, and dose-dependent responses to glycosides **4**–**6** were observed at concentrations of 1–50 nM. All the tested substances exhibited notable synergistic effects with trace amounts of nerve growth factor (NGF, 1 ng/mL) or brain-derived neurotrophic factor (BDNF, 0.1 ng/mL). Using NB cells and OHSCs, it was shown for the first time that starfish steroids **1**–**6** act as neuroprotectors against oxygen-glucose deprivation (OGD) by increasing the number of surviving cells. Altogether, these results suggest that neurotrophin-like neuritogenic and neuroprotective activities are most likely common properties of starfish polyhydroxysteroids and the related glycosides, although the magnitude of the effect depended on the particular compound structure.

## 1. Introduction

Neurotrophins, such as nerve growth factor (NGF) and brain-derived neurotrophic factor (BDNF), are essential for development, differentiation, survival and functional maintenance of neurons in the central and peripheral nervous systems [1,2]. Although neurotrophins are expected to have therapeutic potential in the treatment of neuronal injuries [3], they are rather unstable and too large to pass through the blood-brain barrier that limits their use in medicine. Therefore, low molecular weight compounds mimic the activity of neurotrophins and capable to cross blood-brain barrier should be interesting as promising therapeutic agents to treat traumatic or ischemic brain injuries and neurodegenerative diseases. In recent years, several low molecular weight substances from various natural sources have been shown to possess neurotrophic ability [4,5]. Some of the most effective compounds can be proposed as potential drugs.

**Figure 1:**
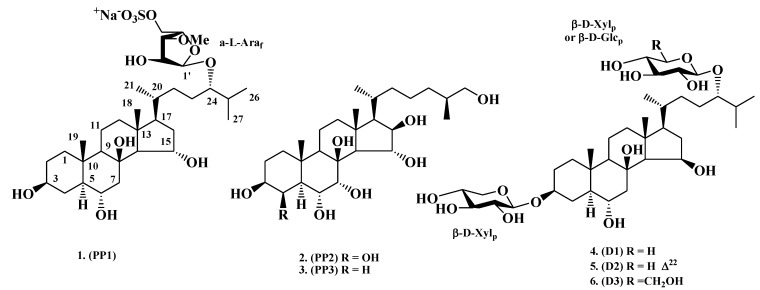
Structures of the compounds **1**−**6**.

Polyhydroxylated steroids and related steroid glycosides are the predominant secondary metabolites in starfish (the phylum Echinodermata, the class Asteroidea) [6,7,8]. Polyhydroxysteroids, as a rule, contain from four to nine hydroxyl groups in steroid nucleus and side chain. Related steroid glycosides have a polyhydroxylated steroid nucleus and one, two or rarely three monosaccharides units attached to polycyclic system, either to side chains or to steroid nucleus and side chain simultaneously. These compounds attract the attention not only because of their peculiar chemical structures but also due to the wide spectrum of their biological activities, including cytotoxic, antiviral, antibacterial, antibiofouling and antifungal effects [6,7,8]. Neuritogenic activity of steroid glycosides from the Okinawan blue starfish *Linckia laevigata* was revealed for the first time by Qi, Han and co-authors [4,9,10] on pheochromocytoma PC12 cells. In our previous work, we demonstrated that polar steroids (polyhydroxysteroids and related glycosides) from various species of starfish could induce neurite outgrowth in mouse neuroblastoma C-1300 cell culture [11,12,13]. The present research is devoted to more detailed study of neurotrophic activities of six starfish steroid compounds: asterosaponin Р_1_, (25*S*)-5α-cholestane-3β,4β,6α,7α,8,15α,16β,26-octaol, and (25*S*)-5α-cholestane-3β,6α,7α,8,15α,16β,26-heptaol, designated as PP1, PP2, and PP3 (**1**–**3**), from *Patiria* (=*Asterina*) *pectinifera* [14], and distolasterosides D_1_–D_3_, designated as D1, D2, and D3 (**4**–**6**), from *Distolasterias nipon* [13] (Figure 1). Besides the investigation of neuritogenic activity of starfish steroids **1**–**6** at low concentrations, we analyzed their neuroprotective ability during oxygen-glucose deprivation using the mouse neuroblastoma C-1300 and organotypic rat hippocampal slice cultures. The starfishes *P. pectinifera* and *D. nipon* are common shallow waters species in the Northwestern Pacific. Compounds **1**–**3** are the most abundant polar steroid constituents in *P. pectinifera* [14], whereas the substances **4**–**6** are the major polar steroid constituents in *D. nipon* [13]*.* Asterosaponin Р_1_ represents the example of monoside with the 3-*O*-methyl-5-*O*-sulfonato-α-l*-*arabinofuranose unit attached to C-24 in a side chain while the both **2** and **3** are free nonsulfated polyhydroxylated compounds. Distolasterosides D_1_–D_3_ (**4**–**6**) are the biosides, containing β-d*-*xylopyranose unit at C-3 and other monosaccharide residue at C-24 (β-d*-*xylopyranose unit in **4** and **5**, and β-d*-*glucopyranose unit in **6**). Moreover, compound **5** have a double bond in the side chain at the position 22(23).

## 2. Results and Discussion

### 2.1. Neuritogenic Activity of Starfish Polar Steroids in Cultured NB Cells

**Figure 2:**
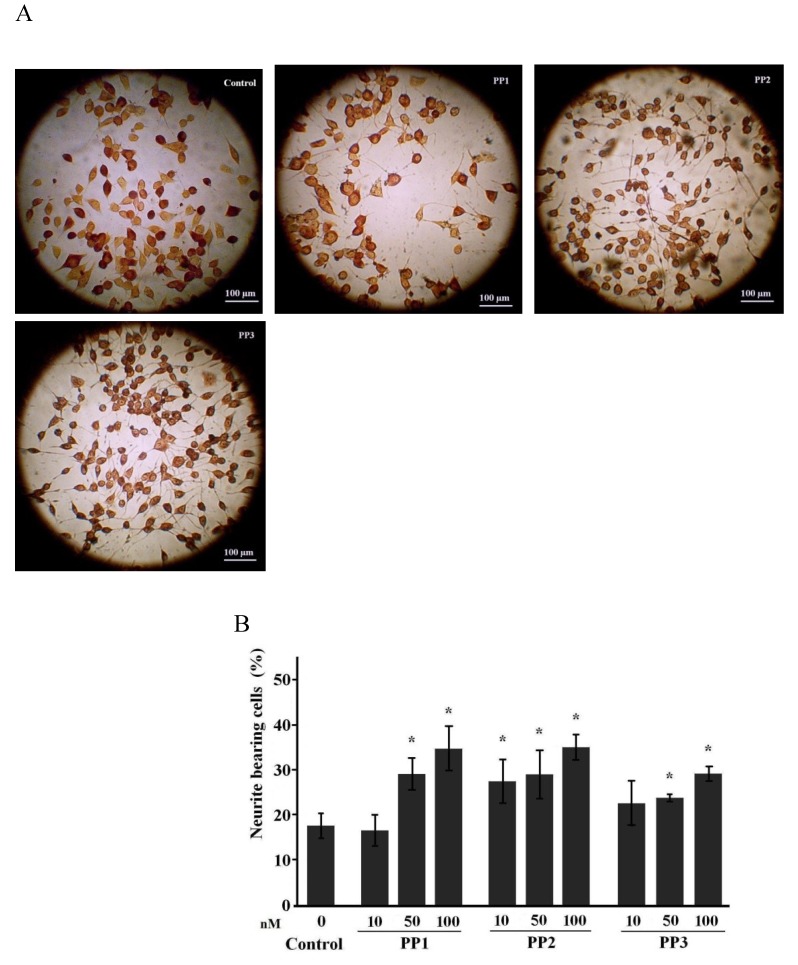
Neuritogenic activity of starfish steroids from *P. pectinifera* in NB C-1300 cells. (**A**) Images of control and steroid-treated cultures. Treatment with compounds PP1, PP2 or PP3 (**1**–**3**) at concentration of 100 nM for 4 days; (**B**) Dose-dependent neuritogenic effects of compounds PP1, PP2, and PP3 (**1**–**3**). The data are presented as the mean values ± SEM from representative experiments. * *p* ≤ 0.05.

**Figure 3:**
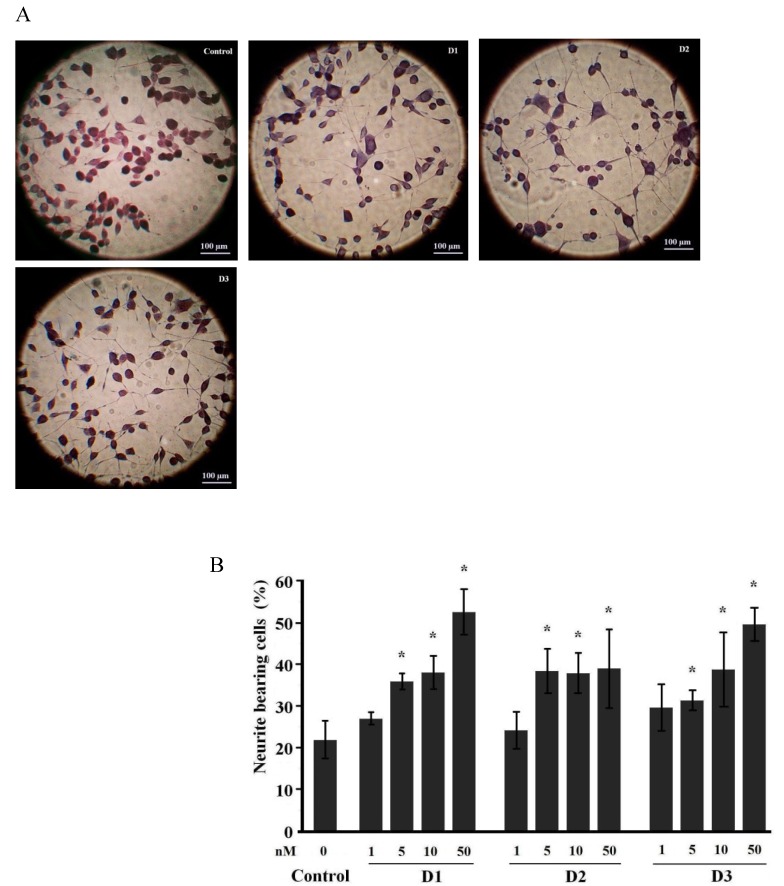
Neuritogenic activity of starfish steroids from *D. nipon* in NB C-1300 cells. (**A**) Images of control and steroid-treated cultures. Treatment with compounds D1, D2 or D3 (**4**–**6**) at concentration of 50 nM for 4 days; (**B**) Dose-dependent neuritogenic effects of compounds D1, D2, and D3 (**4**–**6**). The data are presented as the mean values ± SEM from representative experiments. * *p* ≤ 0.05.

Neuroblastoma (NB) cell cultures are widely used to study neuritogenic and neuroprotective properties of bioactive substances [15,16]. Usually there are several percent of spontaneously differentiating cells in NB C-1300 culture, and the number of differentiated cells increases after prolonged cultivation or cultivation in media with low serum content [17,18]. In our experiments, cells were maintained in 5% bovine fetal serum. The number of cell passages did not exceed 8–10, and each set of experiments contained a control to eliminate mistakes caused by the degree of spontaneous differentiation of the test culture. Thus, in our experiments from 14% to 25% of cells possessed morphological properties of mature neurons (Figure 2B, Figure 3B, controls). It was shown that dimethylsulfoxide (DMSO) also induced the differentiation of NB cells [19,20]. We did not observe significant differences in number of differentiated cells cultured with or without DMSO, possibly due to low final concentration of DMSO. Nevertheless, in all subsequent experiments DMSO was added to control cultures.

The neuritogenic activity of starfish polar steroids in living NB cells can be detected beginning at the second day of cultivation, as we have described previously [11,12,13]. Previous results have demonstrated that a series of polar steroids, including **1**–**3**, manifested significant and nearly identical neuritogenic effects at doses of 2–20 μM, but these effects were slight or even absent at higher concentrations of approximately 40 μM [11]. Polar steroids **4**–**6** from *D. nipon* were effective at lower concentrations (0.1–2 μM) but demonstrated neurotoxic properties at higher concentrations (20–40 μM) [13].

In the present work, we performed more detailed studies on the neuritogenic properties of compounds **1**–**6** at much lower concentrations within 10–100 nM for **1**–**3** and 1–50 nM for **4**–**6** in NB cells using silver-impregnated preparations. Our experiments demonstrated that compounds **1** and **3** increased the number of differentiated neurons (percentage of cells bearing neurites longer than two cell diameters or bearing more than two processes) after four days of incubation at concentrations of 50 nM and higher. Compound **2** had the same effect at doses of 10 nM and above, as did compounds **4**–**6** at doses of 5 nM and above. Dose-dependent responses to all of the compounds were also observed after four days of incubation (Figure 2, Figure 3).

Culturing NB cells in the presence of the starfish steroid compounds also resulted in rapid primary neurite elongation. The mean process lengths ± SEM in the NB cells cultivated with polar steroids **1**–**3** from *P. pectinifera* (at a concentration of 50 nM) were 91.56 ± 5.5 (РР1), 136 ± 7.8 (РР2), and 95.31 ± 4.6 (РР3) μm compared to 105.4 ± 4.3 μm (NGF, 10 ng/mL) and 79.61 ± 4.7 μm in the controls. In the experiments using polar steroids **4**–**6** from *D. nipon* (at a concentration of 50 nM), the process lengths ± SEM were 132.37 ± 8.6 (D1), 119.77 ± 7.19 (D2), and 113.18 ± 7.1 (D3) μm compared to 115.11 ± 2.5 μm (NGF, 10 ng/mL) and 103.94 ± 5.7 μm in the controls. Thus, the neuritogenic effects of the tested compounds in NB cell cultures were comparable to those of NGF. Compound **3** (PP3, 0.5 μM) significantly increased the mean number of processes per cell (2.4 ± 0.2 times) compared to the control (1.7 ± 0.2 times) (*p* ≤ 0.05); a similar effect was not observed in NB cells cultivated with the other tested steroids or NGF.

The ability to stimulate the differentiation of large neurons bearing several processes is a distinct feature of steroids **4**–**6** from *D. nipon* compared to other starfish steroids. Generally, the number of such neurons in self-differentiated NB C-1300 culture is insignificant (Figure 3A).

Qi, Han and co-authors have previously analyzed the biological action of a series of starfish steroid glycosides on pheochromocytoma PC-12 cells [4,9,10]. These cells are not capable of differentiation in the absence of neurotrophic stimuli and respond to NGF by switching from an immature chromaffin cell-like phenotype to a sympathetic neuron-like phenotype characterized the outgrowth of long neurites. In contrast, NB cells are capable of a spontaneous differentiation, and from 14% to 25% of cells were usually differentiated in our experiments. Additionally, NGF appears to stimulate neuritogenesis in only a portion of the neuroblast population in NB cultures. Thus, the differences in the properties of these cell lines underlie the discrepancy in the effective concentrations and the percentage of differentiated cells in our experiments versus the data obtained using PC-12 cells. However, the results obtained using the both cell models confirmed that starfish steroid glycosides and polyhydroxysteroids exhibit evident neuritogenic activities.

**Figure 4:**
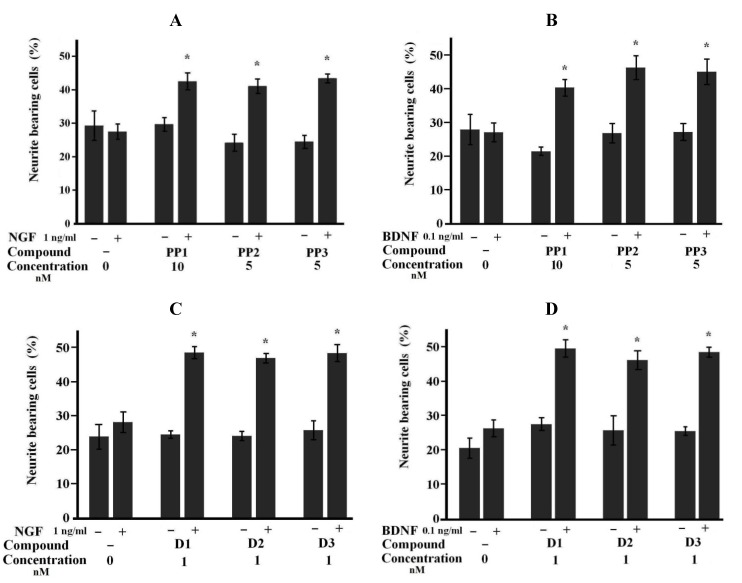
Synergistic effects of neurotrophins and starfish steroids on cell differentiation in NB C-1300 cells. (**A**,**B**) Effects of compounds from *P. pectinifera* and NGF (**A**) or BDNF (**B**). (**C**,**D**) Effects of compounds from *D. nipon* and NGF (**C**) or BDNF (**D**). The data are presented as the mean values ± SEM from representative experiments. * *p* ≤ 0.05.

We also observed that the neuritogenic effects of starfish steroids on NB cells were synergistic with the effects of the neurotrophins NGF or BDNF. Both NGF and BDNF induced the differentiation of NB cells at concentrations of 10 ng/mL; the percentage of neurite-bearing cells reached 46.32% ± 5.79% in NGF-treated and 41.31% ± 3.91% in BDNF-treated NB cells, while in the control, the mean value was 23.64% ± 4.74%. Ineffective (not inducing cell differentiation) concentrations were determined to be 1 ng/mL of NGF and 0.1 ng/mL BDNF. The simultaneous treatment of NB cultures with low concentrations of **1**–**6** and the ineffective concentrations of the neurotrophic factors significantly increased neuronal differentiation (Figure 4).

It is known that some endogenous steroids in mammals stimulate the synthesis of neurotrophins [21,22]. The synergistic effects between the starfish steroids and neurotrophins allow us to assume that the starfish steroids affect the expression of neurotrophins, such as mammalian steroids, or affect the signaling pathways activated by neurotrophins. In fact, it was previously established that the enhancement of NGF-induced neurite outgrowth by granulatoside A, a starfish steroid glycoside, in PC12 cells is attributable to both the increasing and maintenance of phosphorylation of the MAP kinase ERK1/2, although the upstream pathways are unclear [10]. However, it was reported in an article from our group on a related glycoside leviuscoloside G from the starfish *Henricia leviucula* that action of this compound on mouse skin JB6 Cl 41 cells is rather complicated and includes the inhibition of ERKs, NF-κB and AP-1 activities [23]. We suggest that detailed mechanisms underlying the neuritogenic activities of steroid compounds from starfish including their action on all main MAP kinases and different nuclear factors should be investigated and have a plan to participate in these studies in the future.

### 2.2. Neuroprotective Activities of Starfish Polar Steroids in Oxygen-Glucose Deprivation Experiments *in Vitro*

The neuroprotective effects of starfish polar steroids were assessed using NB C-1300 and organotypic hippocampal slice cultures. Both models are used in studies of the protective abilities of different drugs during oxygen-glucose deprivation (OGD) [15,24,25,26]. The OGD period appropriate for different neuroblastoma cell lines usually varied between 3 h up to 24 h depending on the cell line sensitivity [27,28]. Therefore, the time required for the most pronounced OGD effect in NB cells was determined in a preliminary set of experiments. We obtained reproducible results using NB C-1300 cell cultures that were exposed to OGD for 20 h. In this case, the amount of surviving cells was approximately 14%–20%, which was in good agreement with the data obtained using human neuroblastoma cell lines [27,29]. In control NB cultures without OGD treatment the percentage of living cells was approximately 85%–90%. DMSO did not affect the cell surviving in control and OGD-treated cultures.

**Figure 5:**
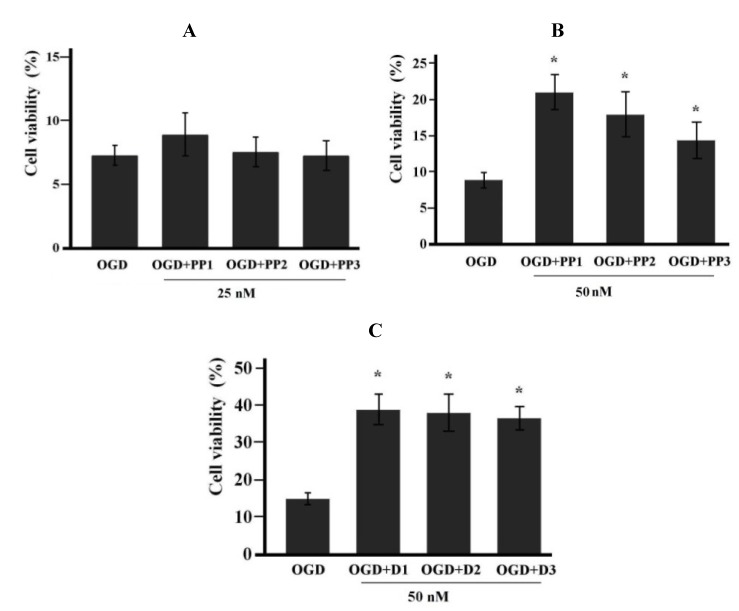
Starfish steroids protect NB C-1300 cells from oxygen-glucose deprivation (OGD). Cultures were maintained in oxygen- and glucose-free medium for 20 h, and the cell viability at 24 h after OGD was determined by counting the cells including trypan blue dye as the percentage of total cells. Starfish steroids were added twice to the cultures at the indicated concentrations for the OGD period and for 24 h after OGD. The data are presented as the mean values ± SEM from representative experiments performed in triplicate. * *p* < 0.05. (**A**) Compounds PP1, PP2, and PP3 (**1**–**3**) failed to increase cell viability at the concentration of 25 nM; (**B**) Protective effects of compounds PP1, PP2, and PP3 (**1**–**3**) at the concentration of 50 nM; (**C**) Protective effects of compounds D1, D2, and D3 (**4**–**6**) at the concentration of 50 nM.

Some differences in the protective abilities of steroids **1**–**3** from *P. pectinifera* and **4**–**6** from *D. nipon* were observed. Although protective effects were not observed for steroids **1**–**3** from *P. pectinifera* at the concentration of 25 nM, reliable neuroprotective properties were detected for the same compounds at a concentration of 50 nM (Figure 5A,B). However, the efficiency of protection at higher concentrations of these steroids, up to 2 μM, did not exceed the values established at a concentration of 50 nM. Steroids from *D. nipon* were more toxic and reduced the number of surviving cells in OGD-treated NB cells at concentrations of 0.5 or 2 μM (data not shown). However, at concentration of 50 nM, these substances showed significant neuroprotective effects (Figure 5C).

Recently, a hypoxia-inducible angiogenic peptide known as vascular endothelial growth factor (VEGF) was identified as a neuroprotector in studies on OGD in NB cell lines [27]. Interestingly, the neuroprotective effects of the polar starfish steroids were comparable to those elicited by VEGF. Possibly, the neuroprotective properties of the starfish steroids could result from their ability to induce cell differentiation. In several recent studies, the relationship between hypoxia and the stage of cell differentiation in solid NB tumors and NB cell lines has been established [30,31,32]. It was demonstrated that hypoxia provoked cell dedifferentiation by down-regulating genes associated with neuronal and upregulating genes associated with a neural crest-like phenotype [33,34], thus contributing to the malignancy of the tumor. Glucose deficiency potentiated the effect of hypoxia [35]. In turn, factors that stimulated NB cell differentiation showed protective activities against hypoxic injury [36].

Organotypic hippocampal slice cultures (OHSCs) demonstrate advantages compared with other organotypic systems used *in vitro* to study the protective effects of various substances because they maintain cell architecture, neuronal connections and electric activity, allowing for extended survival studies [37]. In terms of oxygen-glucose deprivation, OHSCs closely mimic the *in vivo* ischemia-induced injuries [25,26]. To study the neuroprotective activity in OHSCs we selected compounds PP1 (**1**), D1 (**3**) and D3 (**6**), because these compounds were some more effective in neuritogenic and neuroprotective assays on NB culture and were available for further studies. OHSC survival in the presence of **1**, **3**, and **6** (1 μM) was estimated visually during cultivation. After 7–8 days of cultivation, the OHSCs were fixed, stained with cresyl violet or impregnated with silver and studied under a light microscope. None of the tested compounds affected the development of hippocampal slices in culture or their adhesion to collagen in the absence of OGD. Slices maintained the normal arrangement of cellular layers and developed a growth area containing migrated fibroblasts and extended processes similar to control cultures. DMSO added to the culture medium in control OHSCs (0.025%) did not affect the development of slices.

**Figure 6:**
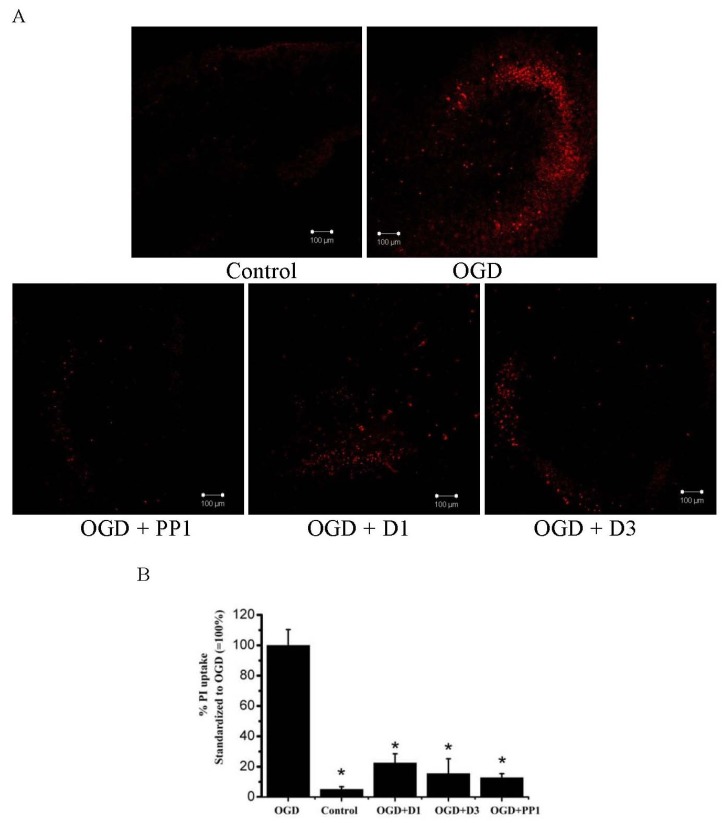
Effect of starfish steroids on cell damage induced by OGD in organotypic hippocampal slice cultures. (**A**) Representative confocal images of control slices and slices stained with propidium iodide (PI) 24 h after exposure to OGD. Starfish steroids were added to OHSCs for 24 h prior to and for 24 h after OGD; (**B**) Quantitative assay of cell damage 24 h after exposure to OGD. Five independent experiments were carried out. The data are presented as the mean values ± SEM. *—a significant difference from exposure to 35 min OGD, *p* ≤ 0.05.

Only single dead cells were present in the control experiments when the OHSCs were stained with propidium iodide (Figure 6A), while massive cell death in various hippocampal areas and fascia dentata was observed in OHSCs exposed to OGD (Figure 6B). There was no significant difference in the amount of cell death OGD-treated and OGD + DMSO-treated cultures. That is in agreement with the previously reported data [26,38,39]. Compounds PP1 (**1**), D1 (**4**) and D3 (**6**) at a concentration of 1 μM were tested in OHSCs under OGD conditions. These starfish steroids reduced the number of propidium-labeled dead cells (Figure 6A,B), providing evidence of neuroprotective properties similar to those of endogenous mammalian steroids previously analyzed using OGD-treated OHSCs [26,38]. The protective effects of mammalian steroids, in particular female sex hormones, have been widely reported in the different types of neuronal cells against various toxicities, including serum deprivation, oxidative stress, amyloid-β peptide, and excitotoxicity [40]. The ability of estrogens to decrease ischemia/reperfusion injury was shown not only in OHSCs [26,38], but in animal models also [41,42]. Although estrogens are neuroprotective, the hormonal effects limit their clinical application, and the search of their analogues with neuroprotective function but lacking hormonal properties is actual. It was shown that the neuroprotective effects of sex hormones could result from their influence on the expression and/or function of neurotrophins [22,43].

Starfish polar steroids, namely, polyhydroxysteroids and related mono- and biglycosides, differ structurally from known protective steroids in mammals. Nevertheless, starfish steroids exhibit the similar neuroprotective abilities and their neuritogenic effects are synergic to neurotrophins. The molecular mechanisms of their effects remain to be investigated.

## 3. Experimental Section

### 3.1. Reagents and Tested Compounds

Dulbecco’s modified Eagle’s medium (DMEM), Iscove’s modified DMEM (IMEM), RPMI 1640 medium, Hank’s balanced solution, penicillin, streptomycin, NGF, BDNF, dimethylsulfoxide (DMSO) and the antimitotic drugs were obtained from Sigma-Aldrich; heat-inactivated fetal bovine serum was obtained from BioloT (St. Petersburg, Russia). The mouse neuroblastoma C-1300 cell line was a kind gift from Professor A.S. Holansky (Institute of Human Morphology, the Russian Academy of Medical Sciences, Moscow, Russia). Compounds **1**–**3** were isolated from the starfish *Patiria* (=*Asterina*) *pectinifera*, and compounds **4**–**6** were isolated from the starfish *Distolasterias nipon*, as described previously, and were pure according to NMR, MS, TLC, and HPLC data [13,14]. Starfish steroids were dissolved in DMSO as stock solutions (4 mM) and then diluted by culture media.

### 3.2. NB Cell Culture

NB C-1300 cells were grown in culture medium containing DMEM:IMEM (3:1), 5% fetal bovine serum, 50 U/mL penicillin, and 50 μg/mL streptomycin at 36 °C in a 90% humidified atmosphere containing 5% CO_2_. The cells were passaged every 2–3 days.

### 3.3. Neuritogenic Effects in NB Cell Cultures

The assays for neuritogenic activity in live cells were performed according to previously described methods [11,12,13]. For experiments involving silver impregnation, the cells were seeded in 6-well plastic plates (2 × 10^4^ cells per well) with glass coverslips. The cells were cultivated overnight, and the medium was replaced with that containing 2% fetal bovine serum and different concentrations of the tested compounds. DMSO in equal concentration was added to culture medium in control. At day 4 of incubation, the cultures on coverslips were fixed with bromformol for 24 h at room temperature and impregnated with silver according to routine protocols. The silver-impregnated preparations were assayed under a Biolar D light microscope (WZO, Poland). Digital images were used for analysis of the neuritogenic effects using the Image Tool program (Union D) according to 3 parameters: % of cells bearing processes at least 2× the cell body diameter or bearing more than 2 processes, the number of neurites per cell, and the length of the primary neurites. In the latter case, the measured segments were summated and converted to μm using an appropriative conversion factor. A total of >100 cells were examined in 10 randomly chosen fields in 4 slides for each concentration of a compound, and 3 independent sets of experiments were conducted for each compound. Data are presented as the means ± SEM. Comparisons between the control and steroid-treated groups were performed using Student’s *t*-tests. A *p*-value less than 0.05 was considered statistically significant.

### 3.4. Oxygen-Glucose Deprivation in NB Cell Cultures

To induce OGD, NB cells in the logarithmic phase of growth were seeded in 24-well plates (2 × 10^4^ cells per well in 3 mL) in serum-free RPMI 1640 medium without glucose that was previously aerated with a 95% nitrogen–5% CO_2_ gas mixture for 1–1.5 h [15]. The plates were placed to a special chamber aerated with the same gas mixture for 20 h at 36 °C and 90% humidity. The cultures were then returned to a normoxic environment for 24 h. Control cultures were cultivated in RPMI 1640 medium with all necessary supplements in a 90% humidified atmosphere containing 5% CO_2_ at 36 °C. The steroid compounds were added to the culture medium twice for the OGD period and subsequent normoxic culture period. DMSO in equal concentration was added to culture medium in control. Cell survival was assessed by adding 0.08% trypan blue dye to the culture wells for 5 min at 25 °C, replacing with dye-free buffer, and counting the dye-containing (injured) and dye-excluding (viable) cells in five 40× microscope fields per well; in most cases, approximately 200 cells per well were counted. Three independent sets of experiments were conducted for each concentration of a compound. The data are reported as the means ± SEM. Comparisons between the control and steroid-treated groups were performed using Student’s *t*-tests. A *p*-value of less than 0.05 was considered statistically significant.

### 3.5. Organotypic Hippocampal Slice Culture

Organotypic hippocampal slice cultures were prepared according to previously described protocols [44]. Briefly, Wistar rats (postnatal day 7–8) were decapitated, and their brains and hippocampi were rapidly removed under aseptic conditions and placed into ice-cold Hank’s balanced solution. The hippocampi were placed into agarose blocks and cut rapidly with a tissue chopper into 400 μm transversal slices. The slices were transferred to collagen-coated coverslips and placed into Petri dishes containing specialized pedestals. Hank’s solution was placed on the bottom of the Petri dishes to supply additional humidity. One hundred microliters of culture medium, consisting of 25% Hank’s balanced solution, 65% DMEM, and 10% fetal bovine serum, was added to each culture. The OHSCs were transferred to a CO_2_-incubator and maintained in a 90% humidified atmosphere with 5% CO_2_ at 36 °C. At day 3 of incubation, antimitotic drugs (5-fluoro-2-deoxyuridine, cytosine-β-d-arabinofuranoside, and uridine, all from Sigma-Aldrich) were added to the culture medium to final concentrations of 1−0.1 μM for 24 h. After the removal of the antimitotics, the OHSCs were cultivated in standard culture medium (control group) or were treated with the steroid compounds at a final concentration of 1 μM. DMSO in equal concentration (0.025%) was added to culture medium in control group. The medium was changed twice a week; the state of the OHSCs was controlled visually. To determine the effects of tested compounds on OHSC development after 7–10 days of cultivation, the cultures were fixed with 4% paraformaldehyde and stained with cresyl violet or impregnated with silver according to routine methods. All experimental procedures involving rats were approved by the Institutional Animal Care and Use Committee and performed according to the Directive 2010/63/EU.

### 3.6. Oxygen-Glucose Deprivation Assay in OHSCs

To analyze protective effects, the steroid compounds at the final concentration of 1 μM were added to the culture medium on day 8 of cultivation. After 24 h, the cultures were rinsed twice with RPMI 1640 medium without glucose that had been aerated previously with a 95% nitrogen–5% CO_2_ gas mixture and treated with the same medium for 35 min in a chamber containing 95% nitrogen–5% CO_2_. Then, the coverslips with OHSCs were rinsed twice with standard medium, placed into Petri dishes, and 100 μL of culture medium containing the examined steroid compounds was added. The cultures were returned to the CO_2_-incubator. In control OHSCs, the standard culture medium was replaced with RPMI 1640 containing glucose for 35 min and then again replaced with standard medium. After 24 h, the OHSCs were stained with propidium iodide (PI) (3 μg per 1 mL of culture medium, 60 min), rinsed with Hank’s balanced solution, fixed with 4% paraformaldehyde (60 min) and embedded in glycerol. Finally, the OHSCs were analyzed with a LSM510 confocal laser scanning microscope (Carl Zeiss, Germany). Propidium iodide was excited with monochromatic light at 543 nm, and images were generated using a diachronic beam splitter (FT 488/543) and a BP 585–605 nm emission bandpass filter. In order to quantify cell death, the optical density of PI fluorescence was recorded. The representative images were captured at the same exposure and digital gain settings to eliminate confounds of differential background intensity or false-positive fluorescent signal across sections. PI fluorescence was quantified using NIH ImageJ software (version 1.46). Cell death area of the 35 min OGD-exposed OHSCs was considered 100%. Compared with this 100% value, cell death in starfish steroids-treated hippocampal slices was calculated. Statistical comparison between OGD and the starfish steroid treatments was done by oneway ANOVA. Five independent experiments were carried out. The data was expressed as a mean ± SEM. Differences with a *p*-value ≤ 0.05 were considered statistically significant.

## 4. Conclusions

Polar starfish steroids, analyzed in the present study, manifest neuritogenic abilities in NB cell cultures, and these effects were synergistic with the actions of NGF and BDNF. They also acted as neuroprotectors in oxygen-glucose deprivation conditions in NB cells and hippocampal slice cultures. Altogether, these results suggest that neurotrophin-like neuritogenic and neuroprotective activities are most likely common properties of starfish polyhydroxysteroids and the related glycosides, although the magnitude of these effects depends on the specific compound structures. The molecular mechanisms of these effects remain to be determined, but polar starfish steroids appear to be promising candidates for further investigation as potential neurotrophic and neuroprotective therapeutics.
